# Titi monkey neophobia and visual abilities allow for fast responses to novel stimuli

**DOI:** 10.1038/s41598-021-82116-4

**Published:** 2021-01-28

**Authors:** Allison R. Lau, Mark N. Grote, Madison E. Dufek, Tristan J. Franzetti, Karen L. Bales, Lynne A. Isbell

**Affiliations:** 1 Animal Behavior Graduate Group, University of California, Davis, Davis, CA 95616 USA; 2 California National Primate Research Center, University of California, Davis, Davis, CA 95616 USA; 3 Department of Psychology, University of California, Davis, One Shields Avenue, Davis, CA 95616 USA; 4 Department of Anthropology, University of California, Davis, One Shields Avenue, Davis, CA 95616 USA; 5 Department of Biology, Duke University, Durham, NC 27708 USA

**Keywords:** Anthropology, Biological anthropology, Animal behaviour

## Abstract

The Snake Detection Theory implicates constricting snakes in the origin of primates, and venomous snakes for differences between catarrhine and platyrrhine primate visual systems. Although many studies using different methods have found very rapid snake detection in catarrhines, including humans, to date no studies have examined how quickly platyrrhine primates can detect snakes. We therefore tested in captive coppery titi monkeys (*Plecturocebus cupreus*) the latency to detect a small portion of visible snake skin. Because titi monkeys are neophobic, we designed a crossover experiment to compare their *latency to look* and their *duration of looking* at a snake skin and synthetic feather of two lengths (2.5 cm and uncovered). To test our predictions that the *latency to look* would be shorter and the *duration of looking* would be longer for the snake skin, we used survival/event time models for *latency to look* and negative binomial mixed models for *duration of looking*. While titi monkeys looked more quickly and for longer at both the snake skin and feather compared to a control, they also looked more quickly and for longer at larger compared to smaller stimuli. This suggests titi monkeys’ neophobia may augment their visual abilities to help them avoid dangerous stimuli.

## Introduction

Over the course of evolutionary history, natural selection has favored the evolution of systems that process signals and cues through an array of sensory modalities^1^. Often, animals become specialists in one or a few sensory modalities. One sensory modality is vision, and among mammals, a distinguishing characteristic of primates is their emphasis on vision as the main sensory modality^2^. An assessment of the visual capabilities of primates suggests there has been especially strong selection for them to see clearly that which is close by and in front of them^3–5^. The selective factors that have driven their visual specialization are hypothesized to revolve around either food resource detection^3,6,7^ or predator avoidance^4,5^.

The idea that primate visual specialization evolved in response to their predators has focused on snakes, in particular. The Snake Detection Theory (SDT) argues that, as the first of the major predators of primates, snakes were largely responsible for primates’ exceptional visual ability. While other predators are best detected and avoided from a distance, it is only necessary to detect and avoid snakes when they are close by^4,5^. Many studies are consistent with or support the SDT (although there are also some that have argued against it:^8,9^). Field studies have documented the apparent awareness of many primate species that snakes are potentially dangerous, as indicated by vocalizations given in the presence of snakes, sustained visual attention, mobbing behavior, and sometimes lethal attacks^10–12^. Vocalizations elicited by snakes draw conspecifics to the location of the snakes, allowing them to learn about the presence of a potential danger and become more vigilant themselves^13–15^. In addition, laboratory studies using different methods have consistently shown that both human and non-human primates visually detect snakes more quickly or attend to them longer than other stimuli, including animate stimuli such as spiders, frogs, caterpillars, and birds [^16–22^; earlier studies reviewed in^23^]. Images of snakes also stimulate more neuronal activity as well as faster and stronger responses in an area of the macaque brain involving attention compared to images of raptors and felids, the two other main classes of predators of primates^24^. While raptors and felids share body plans with other birds and mammals, respectively, the body plan and scale pattern of snakes are shared by no others, perhaps making it possible for selection to favor fast and automatic or non-conscious visual detection of snakes but not other predators, as the SDT argues. Indeed, rapid detection and recognition of snakes appears to be facilitated by their curvilinear shape and their scales^25–30^.

While there is now extensive evidence that primate visual systems hold snakes in a privileged position, the majority of studies have been conducted on humans and other catarrhine primates, such as macaques (*Macaca* spp.) and vervet monkeys (*Chlorocebus pygerythrus*). As catarrhine and platyrrhine primates were exposed to venomous snakes for different amounts of evolutionary time^5^, it is important to broaden the investigation to include more platyrrhines. While several platyrrhine species are reactive toward snakes^26,31–34^, to date, there have been no studies on their latency to detect snakes. Here we investigate the ability of coppery titi monkeys (*Plecturocebus cupreus*) to detect quickly a small portion (2.5 cm) of a snake skin, which provides only the visual cue of scale pattern, and the entire body of a snake, which, in addition to scale pattern, provides the visual cue of a curvilinear shape. Titi monkeys are small-bodied, pair-bonded, platyrrhine primates^35,36^ that are vulnerable to snakes^33,34^ and, like many other primate species, give alarm calls and mob snakes when they are detected^34^.

## Methods

### Subject housing and recruitment

Sixteen titi monkey families underwent testing. Each family had an average of 2.6 animals, with a minimum of 2 animals (N = 10, pair-bonded male and female) and a maximum of 5 animals (N = 1, pair-bonded male and female with 3 offspring). All titi monkeys lived at the California National Primate Research Center (CNPRC)^37^. All study subjects were captive-born and naïve to snakes. The animals were housed in cages measuring 1.2 m × 1.2 m × 2.1 m or 1.2 m × 1.2 m × 1.8 m. The rooms where they were housed were maintained at 21 °C on a 12-h light cycle with lights on at 06:00 h and lights off at 18:00 h. Subjects were fed twice daily on a diet of monkey chow, carrots, bananas, apples, and rice cereal. Water was available ad libitum and additional enrichment was provided twice a day. This setup was identical to housing situations described in previous experiments on this titi monkey colony^37,38^.

Animals were recruited for the study based on availability. Groups with infants younger than 4 months old were excluded from our study. All animals were tested in their family groups.

### Testing design

This study was loosely modeled after a field experiment on latency to detection in which vervet monkeys were exposed to 2.5 cm of a gopher snake skin (*Pituophis catenifer*) stuffed with cotton to give the snake skin a rounded, life-like shape^29^. We used the same gopher snake skin in our study and presented the titi monkeys with the same amount of snake skin, but since titi monkeys are known to be strongly neophobic^39–41^, we also exposed them to 2.5 cm of a blue synthetic feather to determine if their response to the snake skin was a response to a perceived potential danger or simply to a new stimulus in their environment. We used a blue feather because their ability to see blue hues is unaffected by their dichromatic color vision^42^. We predicted that the monkeys’ *latency to look* would be shorter, and the *duration of looking*, longer, for the snake skin than for the feather.

Because the responses to the 2.5 cm snake skin were weak, we later added to the experimental design the entire snake skin but without the head, and the entire feather, to test if a larger snake skin would elicit a stronger response than the partial snake skin. Thus, our final experimental design included four stimuli: 2.5 cm feather, 2.5 cm snake skin, entire feather, and entire snake skin.

### Transport and behavioral testing

**Figure 1:**
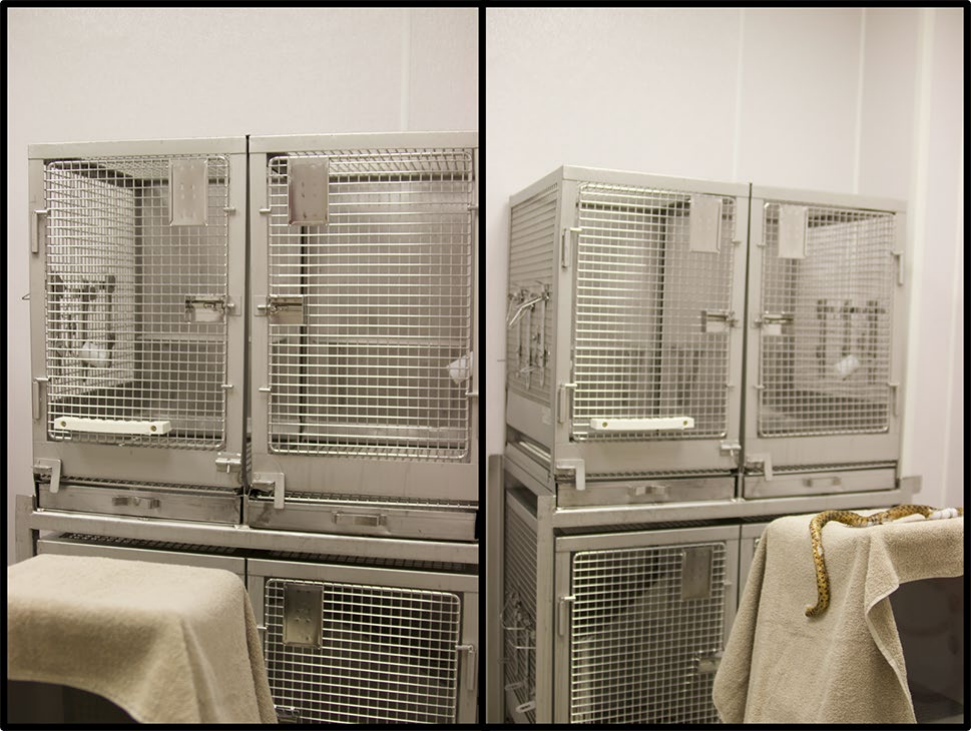
The test cage and platform during the sham control trial and full snake stimulus trial.

All animals were caught in their home cage and transported to the testing room in familiar transport boxes (31 × 31 × 33 cm). We tested them in a separate room from where the animals were housed to ensure other animals in the colony remained naïve to the novel stimuli. We released the animals into a testing cage (Fig. 1) that was baited with Spanish peanuts (1 per monkey) to encourage exploration of the testing cage. Upon transfer of the last monkey, we moved a stimulus platform into view of the animals (approximately 45 cm from the front of the cage). The platform was covered in a tan-colored towel (Fig. 1). Two researchers sat in chairs to the side of the testing setup to score behavior and facilitate the test. The towels covering the test platform and stimuli (described below) were also tan to minimize contrast with the floor and walls.

We used a within- and between-subjects crossover design for this experiment. The order in which subjects saw the test stimuli was counterbalanced across families. Each 20-min testing session consisted of three trials: acclimation, sham control, and stimulus. In the acclimation trial, animals were given ten minutes to acclimate to the new cage, room, and testing platform. No behaviors were scored during this trial. All Spanish peanuts were eaten during this period, and so did not affect subsequent trials.

Once the acclimation trial was over, a research assistant walked between the testing cage and stimulus platform, blocking the stimulus platform with their body. The top towel was lifted and removed, revealing an identical towel below it; then the 5-min sham trial began. This sham trial was used to control for the novelty of manipulating the stimulus platform. This trial will be referred to as “control” in analyses below; all control data were pooled and assessed as one condition.

**Figure 2:**
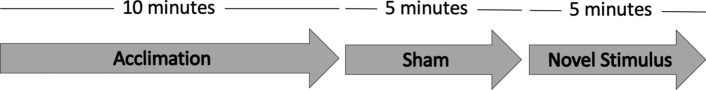
Testing timeline. The novel stimulus presented was a 2.5 cm feather, 2.5 cm snake, entire feather, or entire snake depending on the test condition.

Once the sham control trial ended, the researcher again walked between the testing cage and stimulus platform. The next towel was removed, revealing the test stimulus (Fig. 2). Depending on the test condition, the animals were shown for 5 min either two hand towels covering both ends of the stimulus, leaving 2.5 cm of the stimulus showing, or a fully uncovered stimulus.

On the first day of a 2-day testing period, animals were either shown 2.5 cm of the snake skin or feather during the test trial. Half of our subjects were shown the 2.5 cm feather on the first day, while the other half were shown the 2.5 cm snake. On the second day, animals were shown 2.5 cm of the alternative stimulus type. After a waiting period of at least two weeks, animals participated in another 2-day testing period in which they were either shown the entire feather or entire snake skin during the test trial. Half of our subjects were shown the entire feather on the first day, while the other half were shown the entire snake. On the second day, animals were shown the entire extent of the alternative stimulus type. At the end of testing each day, families were returned to their home cage and monitored for any signs of distress, none of which were observed.

The data from three families that were exposed to the 2.5 cm feather and 2.5 cm snake were not included in the analyses because we collected behavioral data on them via video as part of our pilot testing instead of live-scoring as we did for the other families. These families contributed data only from the entire snake and entire feather test conditions. One family lost a family member partway through the study (unrelated to this study) and thus the surviving family members did not participate in the entire feather or entire snake test conditions.

### Behavior scoring and focal recruitment

During all trials, the *latency to look* (in seconds) at the sham control platform or stimulus was recorded for every family member, including offspring (N = 16 families, N = 40 individuals) by the second observer (MED or TJF). The number of observations for each trial varied based on the number of animals in each family and which stimulus they saw.

Since we did not know which individual would detect the snake skin first, on the first day of testing, one adult from each family was randomly chosen as the focal animal. We observed this animal for the acclimation and sham control trials on that day. The first adult to detect the stimulus during the test trial on the first day then became the focal animal for the test trial and all subsequent trials. One observer (ARL) live-scored *latency to look* and *duration of looking* (also in seconds) for the focal animal using Behavior Tracker 1.5 software (www.behaviortracker.com) on a Dell laptop. The second observer (MED or TJF) was responsible for removing towels between trials, operating a stopwatch, and recording the *latency to look* for non-focal animals. We operationally defined a “look” as both head and eye orientation toward the stimulus that lasted for more than one second in duration. *Look duration* was scored by one observer to ensure consistent scoring. The focal animal’s *latency to look* was scored by both the primary and secondary observer as a test of the primary observer’s reliability. Observers agreed > 95% of the time in scoring *latency to look* for the same animals. Observers were not blind to condition since the stimulus platform was visible to all subjects and observers.

### Data analysis

We used a survival/event time model for the response variable *latency to look* because a few animals did not respond to the stimulus during the 5-min test trial and therefore had censored observations of *latency to look* (N = 15 observations across 11 unique individuals from 10 different families). We fitted two Cox Proportional Hazards regression models using the *coxph* function of the *survival* library^43^ invoked from the R statistical computing language^44^. The first was a null model including “cluster robust” standard errors to accommodate repeated measures on each subject. The second model added main effects of experimental condition and stimulus order, along with their interaction, to capture the structure of the crossover experiment. The interaction of experimental condition and stimulus order allowed for the effect of the stimulus to depend on the order in which the stimuli were shown. Model comparison of the second model to the first, using Akaike’s Information Criterion (AIC), assessed the extent to which animals responded to the experiment. To check the fit of the models, we examined a graph of the cumulative hazard function of the Cox-Snell residuals, e.g., [^45^:356].

The response variable *duration of looking* (seconds) was integer-valued and highly variable across subjects, suggesting that a negative binomial model would be appropriate. We fitted two generalized linear mixed-effects models using the *glmmadmb* function of the *glmmADMB* library^46^. The first was a null model incorporating random intercepts to accommodate repeated measures on each subject. The second model added effects of experimental condition and stimulus order as above for *latency to look*. We examined a quantile–quantile plot of the Pearson residuals compared to a chi-squared distribution with one degree of freedom to assess goodness of fit, and compared the first and second models using AIC.

We tested eight planned contrasts to infer the effects of stimulus length and stimulus type on our animals’ *latency to look* and *duration of looking*. Under the crossover design, stimuli were necessarily presented to each family in a given order. Although presentation order was counterbalanced across families, we nonetheless wished to contrast stimuli in a manner that was, broadly speaking, indifferent to order. Marginalizing model estimates with respect to order, described in detail in supplemental material 1, meets this need. We calculated marginal contrasts and confidence intervals for the following: entire snake vs. entire feather, 2.5 cm snake versus 2.5 cm feather, entire snake versus 2.5 cm snake, and entire feather versus 2.5 cm feather for both of the response variables, *latency to look* and *duration of looking*. We used the Bonferroni correction for eight comparisons to preserve a 5% study-wide false positive rate. We estimated marginal contrasts and standard errors using the log scale of the Cox Proportional Hazards and negative binomial models.

### Ethical note

This study was approved by the IACUC of the University of California, Davis. This study met all legal requirements of the United States as well as guidelines set by the American Society of Primatologists for the ethical treatment of non-human primates. This study was carried out in compliance with the ARRIVE guidelines.

## Results

### Latency to look

**Figure 3:**
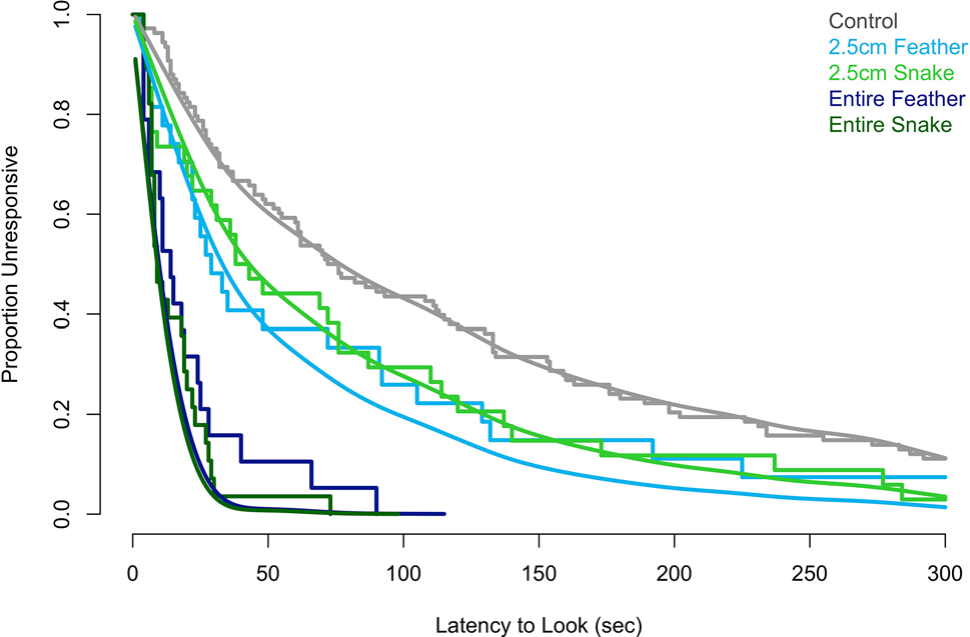
Empirical Kaplan–Meier survival/event time curves and model predictions for *latency to look* (s) for 40 titi monkeys in response to five stimulus types. Empirical curves were produced by the *survfit* function from the *survival* library^43^ and are shown as stair-step lines, while model predictions are overlaid as smooth curves. Color denotes the stimulus type.

We included *latency to look* for 216 observations from all 40 individuals across 16 families. The number of observations per individual was variable depending upon how many test conditions each monkey saw (reasons for not completing all four test conditions are included in the methods). Subjects’ *latency to look* (seconds) was recorded for the sham control [median (25th, 75th percentiles): 74 (28, 176)], 2.5 cm feather [29 (16, 99)], 2.5 cm snake [41 (12, 113)], entire feather [14 (6, 25)], and entire snake [9 (7, 21)]. Based on model comparisons with the AIC, the model for *latency to look* incorporating experimental effects was preferred over the null model [AIC(null) = 1842, AIC(experimental) = 1784]. Broadly speaking, the experiment had strong consequences for *latency to look*. Based on the survival/event time curves (Fig. 3), animals typically attended more quickly to the 2.5 cm feather, followed by the 2.5 cm snake and the control. The entire snake and the entire feather were both looked at earlier in time than the other three stimuli. Although the smooth, fitted curves in Fig. 3 do not very well distinguish the entire snake and entire feather, the full model fits well overall (see Fig. S.1 in Supplemental Material 1).

### Duration of looking

**Figure 4:**
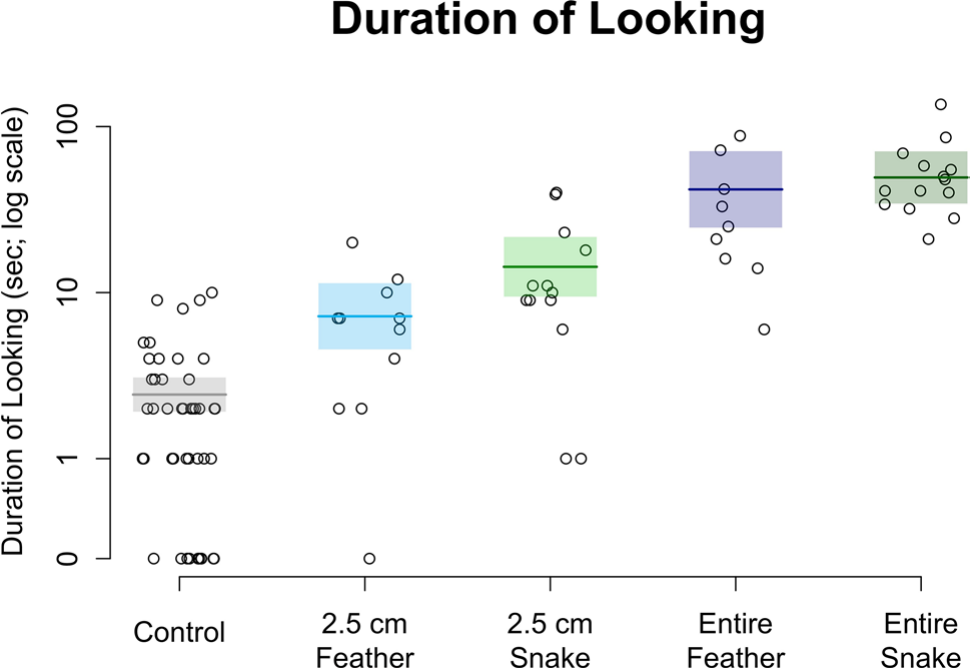
Observed *durations of looking* (s) in response to five stimulus types and model predictions. Predicted durations are from a negative binomial mixed model. Center lines give the predicted means and shaded boxes show one standard error intervals. The vertical axis is on the natural-log scale, though the axis tick marks are labeled according to the original scale of time in seconds. Observations with a duration of zero seconds have been superimposed at the bottom of the vertical axis for completeness.

We included *duration of looking* for 94 observations from 18 individuals across 16 families. We had a total of 18 individuals because for two families, our focal individual changed partway through the experiment due to the focal recruitment criteria described above. Subjects’ *duration of looking* (seconds) was recorded for the sham control [median (25th, 75th percentiles): 2 (1, 3)], 2.5 cm feather [7 (3, 9)], 2.5 cm snake [10 (9, 18)], entire feather [25 (16, 42)], and entire snake [45 (36, 57)]. Based on model comparisons with the AIC, the model for *duration of looking* incorporating experimental effects was preferred over the null model [AIC(null) = 683, AIC(experimental) = 576]. As with *latency to look*, the experiment had strong consequences for *duration of looking*. They were shortest for the sham control, followed by the 2.5 cm feather, the 2.5 cm snake, the entire feather, and finally the entire snake (Fig. 4). Some animals never looked at the control (N = 11 observations from 10 individuals) and one animal never looked at the 2.5 cm feather. All animals spent some amount of time looking at the 2.5 cm snake, the entire feather, and the entire snake.

### Planned contrasts

In order to address our predictions more quantitatively, we planned eight contrasts. Namely, we assessed if animals had a shorter *latency to look* and/or a longer *duration of looking* for potentially dangerous compared to non-dangerous stimuli (four contrasts: 2.5 cm snake vs. 2.5 cm feather, entire snake vs. entire feather for *latency to look* and *duration of looking*). We also assessed if animals had a shorter *latency to look* and/or a longer *duration of looking* for larger compared to smaller stimulus types (four contrasts: entire snake vs. 2.5 cm snake, entire feather vs. 2.5 cm feather for *latency to look* and *duration of looking*).

**Figure 5:**
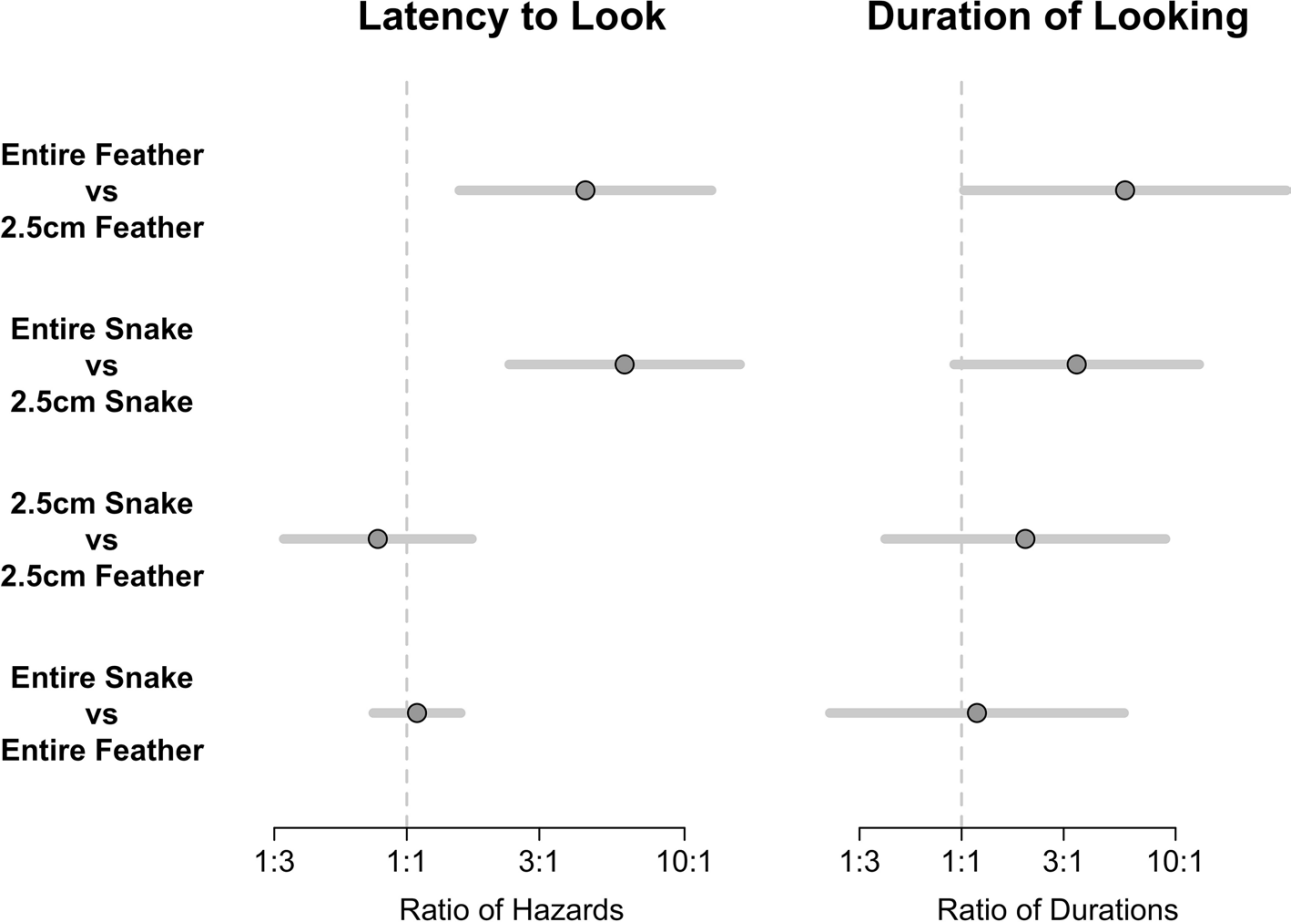
Results from eight planned contrasts assessing *latency to look* and *duration of looking* in response to feather and snake stimuli. Center dots represent the average marginal contrast. For *latency to look*, a center dot to the right of the 1:1 line indicates that the hazard for the first stimulus in the comparison is greater, and therefore that the *latency to look* is shorter. For *duration of looking*, a center dot to the right of the 1:1 line indicates that *duration* for the first stimulus in the comparison is greater. The error bars represent ± 2.73 SE of the marginal contrast, as appropriate for eight planned comparisons. The horizontal axes reflect ratio comparisons of the paired stimuli––hazard ratio for *latency to look* and ratio of durations for *duration of looking*. For example, the hazard for the entire feather was more than three times the hazard for the 2.5 cm feather, on average.

For eight contrasts, the Bonferroni correction required that we construct ± 2.73 SE intervals instead of the usual ± 2 SE interval used to test a single hypothesis at a 5% level. Thus, each interval was relatively conservative. For five of the eight contrasts, the confidence interval contained the null value zero, indicating that the responses to the two stimuli compared were not different enough to be considered statistically significant (Fig. 5). For *latency to look*, the estimated hazard for the full snake was more than three times greater than the hazard for the 2.5 cm snake and this comparison was statistically supported, with a Bonferroni confidence interval that did not intersect the 1:1 comparison line. Thus, the monkeys’ *latency to look* was shorter for the entire snake than the 2.5 cm snake. Similarly, for *latency to look,* the estimated hazard for the full feather was more than three times greater than the hazard for the 2.5 cm feather and this comparison was statistically supported, with a Bonferroni confidence interval that did not intersect the 1:1 comparison line. The ratio of *duration of looking* for the entire feather as compared to the 2.5 cm feather was approximately 3:1 on average, and this comparison was also statistically supported, although the lower confidence limit was only slightly above the 1:1 comparison line (Fig. 5).

## Discussion

Unexpectedly, titi monkeys were relatively unresponsive in the presence of the partial snake skin. Vervets looking at the same snake skin simultaneously engaged in other responses, including bending down and peering, remaining still and staring, and standing bipedally to look at the snake skin^29^. We saw no such responses from the titi monkeys. Moreover, the first titi monkeys to look at the partial snake skin were slower (median: 41 s) than the first (free-ranging) vervets (median: 10 s) with similarly unobstructed views^29^. These muted behavioral responses to the partial snake skin, and the lack of a differential response from titi monkeys toward the snake skin and feather with only 2.5 cm showing might be interpreted as non-recognition of potential danger driven by poorer visual ability to detect fine detail such as scale lines. Alternatively, as the titi monkeys used in this study were three to eight generations removed from the original wild founder population, the lack of a differential response could have been driven by a history of captive rearing without exposure to snakes. This possibility prompted us to test them with the entire snake skin and the entire feather. More attention directed to the snake skin than the feather would suggest captivity had minimal effects on their ability to perceive the snake as a potential threat.

While titi monkeys tended to look more quickly (9 vs. 14 s) and for a longer duration (41 vs. 25 s) at the entire snake than the entire feather, suggesting that captivity has not extinguished their visual attraction to snakes, the most obvious differences, as identified in planned comparisons that did not overlap the 1:1 line, were in the *latency to look* at the entire snake (9 s) versus the partial snake (41 s), the *latency to look* at the entire feather (14 s) versus the partial feather (29 s), and the *duration of looking* at the entire feather (25 s) versus the partial feather (7 s). These results reveal that larger stimuli generated more attention than stimuli reflecting a potential threat. This is in line with the neophobic nature of adult titi monkeys in that they respond with caution and visual orientation to novel objects and conditions much more so than other platyrrhines^39–41^. Coupled with vision, their neophobia should be beneficial to them in detecting and better avoiding dangerous animals. A question to consider for the future is whether their neophobia might be part of an independently evolved strategy in response to the threat from venomous snakes as a form of compensation for poorer vision.

Placing our study in a broader theoretical context, one of the hypotheses of the SDT is that catarrhine primates are uniformly capable of detecting snakes quickly because their common ancestor evolved in the presence of venomous snakes, a selective pressure that has persisted to the present day. Platyrrhine primates, in contrast, are hypothesized to vary more in their ability to detect snakes because they began diversifying prior to the arrival of venomous snakes in South America. Selection thus would have operated on platyrrhine lineages more independently as venomous snakes became established there^4,5^. The lineage leading to titi monkeys, for example, is estimated to have diverged 16–19.5 million years ago^47,48^, before bothropoid snakes began diverging in South America^49,50^. In addition to studies of comparative brain morphology and neural connectivity (discussed in [^5^:103–106]), three lines of behavioral evidence appear to support the hypothesis of greater variability in platyrrhine visual systems:

1. Head-cocking is a behavior that is generated more by novel visual stimuli than by recognition of threat, and may help in discriminating the object of attention^51–54^. No catarrhines head-cock to novel stimuli, whereas platyrrhines are more variable^51^.
2. Catarrhines react to two-dimensional images as they do to three-dimensional images, including snakes^55–67^, whereas platyrrhines are more variable^26,54,55,68–71^.
3. Of all the platyrrhines, capuchin monkey visual systems appear to be most convergent with those of catarrhines^5^. This may also be reflected behaviorally. Capuchins do not head-cock to models of snakes or novel stimuli^26^, they react similarly to two- and three-dimensional images, and, like macaques and humans, they are able to distinguish between dangerous and non-dangerous snakes^26,32,72,73^, an ability that undoubtedly requires excellent visual discrimination. Capuchins are the most terrestrial of platyrrhines, and the risk from terrestrial as well as arboreal venomous snakes may have put a premium on excellent vision for objects that are close by and in front of oneself.

Testing the hypothesis that platyrrhines have greater variability than catarrhines in snake detection requires replicable studies of many taxa of the latency to detect snakes, ideally with primates that have likely had experience with snakes, i.e., under field conditions. We have one other recommendation for future comparative studies. Our study’s planned contrasts of the titi monkey responses limited our ability to detect a signal among the noise of our study design. We designed eight planned contrasts to examine the sensitivity of titi monkeys to partial and entire snakes and used the Bonferroni correction for eight comparisons to preserve a 5% study-wide false positive rate. This correction may have produced more conservative confidence intervals, thus resulting in several null findings. Our statistical corrections may have further muted the reduced response of titi monkeys to snake skins. Future studies should aim for larger sample sizes to minimize any such statistical limitations.

## Supplementary Information

Supplementary Information 1.

Supplementary Information 2.

Supplementary Information 3.

Supplementary Information 4.

## Data Availability

All data generated or analyzed during this study are included in this published article (and its Supplementary Information files).
