# Supplementary material for: Titi monkey neophobia and visual abilities allow for fast responses to novel stimuli
Source: Sci Rep. 2021 Jan 28;11:2578. doi: 10.1038/s41598-021-82116-4 (PMC7844259; doi:10.1038/s41598-021-82116-4)
Supplement: Supplementary file 2 — Supplementary Information 2. [file 41598_2021_82116_MOESM2_ESM.pdf]

```
ave.marginal.effect <- function(model.obj, eff) {
  mu <- coef(model.obj)["(Intercept)"]
  beta <- coef(model.obj)[eff]
  gamma.order <- coef(model.obj)["revised.order"]
  theta.string <- paste(eff,"revised.order", sep=":")
  theta <- coef(model.obj)[theta.string]
  log(0.5*exp(mu+beta)*(1+exp(gamma.order+theta)))
}

se.marginal.effect <- function(model.obj, eff) {
  mu <- coef(model.obj)["(Intercept)"]
  beta <- coef(model.obj)[eff]
  gamma.order <- coef(model.obj)["revised.order"]
  theta.string <- paste(eff,"revised.order", sep=":")
  theta <- coef(model.obj)[theta.string]
  g.prime <- rep(0, length(coef(model.obj)))
  names(g.prime) <- names(coef(model.obj))
  ## we are giving g.prime names interherited from the model object for correct substitution
later on
  g.prime["(Intercept)"] <- 1
  g.prime[eff] <- 1
  g.prime[theta.string] <- exp(gamma.order+theta)/(1+exp(gamma.order+theta))
  g.prime["revised.order"] <- g.prime[theta.string]
  var <- t(g.prime) %*% vcov(model.obj) %*% g.prime
  sqrt(var)
}

ave.marginal.control <- function(model.obj) {
  mu <- coef(model.obj)["(Intercept)"]
  gamma.order <- coef(model.obj)["revised.order"]
  log(0.5*exp(mu)*(1+exp(gamma.order)))
}

se.marginal.control <- function(model.obj) {
  mu <- coef(model.obj)["(Intercept)"]
  gamma.order <- coef(model.obj)["revised.order"]
  g.prime <- rep(0, length(coef(model.obj)))
  names(g.prime) <- names(coef(model.obj))
  ## we are giving g.prime names interherited from the model object for correct substitution
later on
  g.prime["(Intercept)"] <- 1
  g.prime["revised.order"] <- exp(gamma.order)/(1+exp(gamma.order))
  var <- t(g.prime) %*% vcov(model.obj) %*% g.prime
  sqrt(var)
```

```
}

ave.marginal.contrast <- function(model.obj, eff1, eff2) {
  beta1 <- coef(model.obj)[eff1]
  beta2 <- coef(model.obj)[eff2]
  gamma.order <- coef(model.obj)["revised.order"]
  theta1.string <- paste(eff1,"revised.order", sep=":")
  theta2.string <- paste(eff2,"revised.order", sep=":")
  theta1 <- coef(model.obj)[theta1.string]
  theta2 <- coef(model.obj)[theta2.string]
  beta1 + log(1+exp(gamma.order + theta1)) - (beta2 + log(1+exp(gamma.order + theta2)))
}

## standard error of the average marginal contrast
se.marginal.contrast <- function(model.obj, eff1, eff2) {
  beta1 <- coef(model.obj)[eff1]
  beta2 <- coef(model.obj)[eff2]
  gamma.order <- coef(model.obj)["revised.order"]
  theta1.string <- paste(eff1,"revised.order", sep=":")
  theta2.string <- paste(eff2,"revised.order", sep=":")
  theta1 <- coef(model.obj)[theta1.string]
  theta2 <- coef(model.obj)[theta2.string]
  g.prime <- rep(0, length(coef(model.obj)))
  names(g.prime) <- names(coef(model.obj))
  ## we are giving g.prime names interherited from the model object for correct substitution
later on
  g.prime[eff1] <- 1
  g.prime[eff2] <- -1
  g.prime[theta1.string] <- exp(gamma.order+theta1)/(1+exp(gamma.order+theta1))
  g.prime[theta2.string] <- -exp(gamma.order+theta2)/(1+exp(gamma.order+theta2))
  g.prime["revised.order"] <- g.prime[theta1.string] + g.prime[theta2.string]
  var <- t(g.prime) %*% vcov(model.obj) %*% g.prime
  sqrt(var)
}

#Add translucent color function.
addTrans <- function(color,trans)
{
  # This function adds transparancy to a color.
  # Define transparancy with an integer between 0 and 255
  # 0 being fully transparant and 255 being fully visable
  # Works with either color and trans a vector of equal length,
  # or one of the two of length 1.
```

```
  if (length(color)!=length(trans)&!any(c(length(color),length(trans))==1)) stop("Vector lengths
not correct")
  if (length(color)==1 & length(trans)>1) color <- rep(color,length(trans))
  if (length(trans)==1 & length(color)>1) trans <- rep(trans,length(color))

  num2hex <- function(x)
  {
    hex <- unlist(strsplit("0123456789ABCDEF",split=""))
    return(paste(hex[(x-x%%16)/16+1],hex[x%%16+1],sep=""))
  }
  rgb <- rbind(col2rgb(color),trans)
  res <- paste("#",apply(apply(rgb,2,num2hex),2,paste,collapse=""),sep="")
  return(res)
}
```
