# Supplementary material for: Titi monkey neophobia and visual abilities allow for fast responses to novel stimuli
Source: Sci Rep. 2021 Jan 28;11:2578. doi: 10.1038/s41598-021-82116-4 (PMC7844259; doi:10.1038/s41598-021-82116-4)
Supplement: Supplementary file 3 — Supplementary Information 3. [file 41598_2021_82116_MOESM3_ESM.pdf]

```
## Code for Titi monkey neophobia and visual abilities allow for fast responses
## to novel stimuli
## Written by Mark Grote and Allison Lau
## Last updated 11.8.2020

## Read in necessary libraries
library(glmmADMB)
library(survival)
source("/Users/ /Snake Titi Crossover Helper Functions_10.28.2020_revised order.R")

## TABLE OF CONTENTS
## SECTION 0: DATA MANAGEMENT
## SECTION 1: MODEL FOR LOOK DURATION
## SECTION 2: MODEL FOR LATENCY TO DETECTION
## SECTION 3: CALCULATE AND DISPLAY CONTRASTS FOR BOTH MODELS

### SECTION 0: DATA MANAGEMENT

## Read in data file.
SDLdata <- read.csv("/Users/ /expandeddata_long format_10.27.2020.csv", stringsAsFactors =
T)
#SDLdata <- read.csv("finaldata_long format.csv") #Mark's read-in.

## Make "control" the reference level of the factor experiment.
SDLdata$experiment <- factor(SDLdata$experiment,
	levels=c("Control", "1\" Feather", "1\" Snake", "Full Feather", "Full Snake"),
	labels=c("Control", "1in.Feather", "1in.Snake", "FullFeather", "FullSnake"))

## Count the number of incomplete cases (lines with one or more NAs)
sum(!complete.cases(SDLdata))

## Remove animals that have missing latency data.
SDLdata <- subset(SDLdata, subset = !is.na(latency))

### SECTION 1: MODEL FOR DURATION OF LOOKING

## Removing records with missing duration of looking. The animals were not able to participate
for a given
## stimulus.
SDLdata.lookdur <- subset(SDLdata, subset = !is.na(look.duration))

## Null model for look duration having only an overall mean and animal-specific random
intercepts.
```

```
lookdur.m0 <-glmmadmb(look.duration ~ 1 + (1|name), data=SDLdata.lookdur,
family="nbinom", verbose = TRUE)
summary(lookdur.m0)

## Model with interaction of experimental condition and order of experimental condition to
capture the
## structure of the crossover experiment. The interaction of experimental condition and order
allows
## for the effect of the stimulus to depend on the order in which the stimuli were shown.
lookdur.m1 <-glmmadmb(look.duration ~ experiment * revised.order + (1|name),
data=SDLdata.lookdur, family="nbinom",
     verbose = TRUE)
summary(lookdur.m1)

## GOODNESS OF FIT CHECKS FOR m1
lookdur.m1.pearson <- residuals(lookdur.m1, type=c("pearson"))

## Calculate the Pearson statistics, the sum of the squared pearson residuals, and compare to
sample size.
sum(lookdur.m1.pearson^2)

## Residual plot for m1.
lookdur.m1.fitted <- fitted(lookdur.m1)
plot(x= log(lookdur.m1.fitted), y=lookdur.m1.pearson, bty="n", xlab="Predicted Value",
ylab="Pearson Residual",
    main="Residual Plot Model lookdur.m1")

## Quantile-quantile plot of the squared Pearson residuals compared to a chi-squared
## distribution with one degree of freedom.
lookdur.m1.pearson.sq <- lookdur.m1.pearson^2
jpeg(file= "/Users/ /lookduration.goodness.fit.revisedcoding.jpg",
    height=6, width=6, units = "in", res = 500)
plot(qchisq(ppoints(lookdur.m1.pearson.sq),df=1),sort(lookdur.m1.pearson.sq),xlab="Chi-
Square (1) Quantile",
    ylab="Squared Pearson Residual", bty="n", main="Duration of Looking m1 Goodness of Fit")
abline(a=0, b=1)
graphics.off()

## Compare AICs from lookdur.m0 and lookdur.m1.
AIC(lookdur.m0,lookdur.m1)

## GRAPH OF DATA AND PREDICTIONS FROM m1.
SDLdata.lookdur$log.look.duration <-
```

```
  ifelse(SDLdata.lookdur$look.duration > 0, log(SDLdata.lookdur$look.duration),
log(SDLdata.lookdur$look.duration + 0.25))

### Create a data frame of the average marginal effects and confidence intervals for m1.
lookdur.m1.results <- data.frame(
  effect=rep(0,5),
  ll=rep(0,5),
  ul=rep(0,5),
  row.names = c("Control", "experiment1in.Feather", "experiment1in.Snake",
              "experimentFullFeather", "experimentFullSnake")
)

for(eff in row.names(lookdur.m1.results)){
  if(eff!="Control") {
    estimate <- ave.marginal.effect(lookdur.m1, eff)
    se <- se.marginal.effect(lookdur.m1, eff)
    ll <- estimate - se
    ul <- estimate + se
    lookdur.m1.results[eff, 1:3] <- c(estimate,ll,ul)
  }
  else{
    estimate <- ave.marginal.control(lookdur.m1)
    se <- se.marginal.control(lookdur.m1)
    ll <- estimate - se
    ul <- estimate + se
    lookdur.m1.results[eff, 1:3] <- c(estimate,ll,ul)
  }
}

## Define structures for lookdur.m1 results plot.
preds.mean <- lookdur.m1.results$effect
preds.lb <- lookdur.m1.results$ll
preds.ub <- lookdur.m1.results$ul

### Color and graphics parameters for lookdur.m1 results plot.
y.vals <- c(0.5,1.5,2.5,3.5)
my.pt.cex <- 2
my.line.wd <- 5
my.line.cl <- "gray80"
my.pt.col <- "gray80"
my.text.cex <- 1.5
poly.density <- 10
poly.width <- 0.5
my.asp <- 0.4
```

```
my.poly.gray <- "gray60"
my.poly.lightgreen <- "limegreen"
my.poly.lightblue <- "deepskyblue2"
my.poly.darkgreen <- "darkgreen"
my.poly.darkblue <- "darkblue"

## Use helper function to add transparency to polygon shading colors.
my.poly.gray <-addTrans(my.poly.gray, 80)
my.poly.lightgreen <-addTrans(my.poly.lightgreen, 80)
my.poly.lightblue <-addTrans(my.poly.lightblue, 80)
my.poly.darkgreen <-addTrans(my.poly.darkgreen, 80)
my.poly.darkblue <-addTrans(my.poly.darkblue, 80)

### Make graph.
jpeg(file= "/Users/ /lookdur.results.revisedorder.jpg",
    height=6, width=8, units = "in", res = 500)
par(oma=c(1,1,1,0)+0.5)
x.jitter <- jitter(as.numeric(SDLdata.lookdur$experiment), factor=1)
plot(x=x.jitter, y=SDLdata.lookdur$log.look.duration, bty="n", xlab=" ", ylab="Duration of
Looking (sec; log scale)",
    xaxt="n", yaxt="n", cex.lab=my.text.cex)
axis(side=1, at=1:5, labels=c("Control", "2.5 cm\nFeather", "2.5 cm\nSnake", "Entire\nFeather",
"Entire\nSnake"),
    padj=0.75, cex.axis=my.text.cex)
axis(side=2, at=c(log(0.25),log(1),log(10),log(100)), labels=c("0","1","10","100"),
cex.axis=my.text.cex)
title(main="Duration of Looking", cex.main=2, line=1.9)

## Adding the mean to polygons.
segments(x0=c(1 - poly.width/2, 2 - poly.width/2, 3 - poly.width/2, 4 - poly.width/2, 5 -
poly.width/2 ),
       y0=preds.mean, x1= c(1 + poly.width/2, 2 + poly.width/2, 3 + poly.width/2, 4 +
poly.width/2, 5 + poly.width/2),
       col = c("gray60","deepskyblue2", "darkgreen", "darkblue", "darkgreen"),
       lwd = 1.75)

## Polygons for the five arrays.
polygon(x = c(1 - poly.width/2, 1-poly.width/2, 1 + poly.width/2, 1 + poly.width/2),
      y = c(preds.lb[1], preds.ub[1],
          preds.ub[1], preds.lb[1]),
      density=my.poly.gray,
      border=NA,
      col=my.poly.gray)
polygon(x = c(2 - poly.width/2, 2-poly.width/2, 2 + poly.width/2, 2 + poly.width/2),
```

```
        y = c(preds.lb[2], preds.ub[2],
              preds.ub[2], preds.lb[2]),
        density=my.poly.lightblue,
        border=NA,
        col=my.poly.lightblue)
polygon(x = c(3 - poly.width/2, 3-poly.width/2, 3 + poly.width/2, 3 + poly.width/2),
        y = c(preds.lb[3], preds.ub[3],
              preds.ub[3], preds.lb[3]),
        density=my.poly.lightgreen,
        border=NA,
        col=my.poly.lightgreen)
polygon(x = c(4 - poly.width/2, 4-poly.width/2, 4 + poly.width/2, 4 + poly.width/2),
        y = c(preds.lb[4], preds.ub[4],
              preds.ub[4], preds.lb[4]),
        density=my.poly.darkblue,
        border=NA,
        col=my.poly.darkblue)
polygon(x = c(5 - poly.width/2, 5-poly.width/2, 5 + poly.width/2, 5 + poly.width/2),
        y = c(preds.lb[5], preds.ub[5],
              preds.ub[5], preds.lb[5]),
        density=my.poly.darkgreen,
        border=NA,
        col=my.poly.darkgreen)

graphics.off()


### SECTION 2: MODEL FOR LATENCY TO DETECTION

## A few latencies are censored because animals did not respond to the stimulus. These are enough to
## warrant a survival/event time model.

## Use "cluster robust" method of function coxph, in lieu of animal-specific frailty.
## Null model for latencies to detection -- Cox Proportional Hazards Model
lat.m0 <- coxph(Surv(latency, status) ~ 1, cluster=name, SDLdata)
summary(lat.m0)

## Model for  latencies to detection with interaction of experimental condition and order of experimental
## condition to capture the structure of the crossover experiment. The interaction of experimental
## condition and order allows for the effect of the stimulus to depend on the order in which the stimuli
```

```
## were shown.
lat.m1 <- coxph(Surv(latency, status) ~ experiment * revised.order,
          cluster=name, SDLdata, model=TRUE)
summary(lat.m1)

AIC(lat.m0, lat.m1)

## Residual goodness of fit check from
https://www.math.wustl.edu/~jmding/math434/R_model_diag.R
## First get the Cox-Snell residuals. The default residuals of coxph in R are the martingale
residuals.
## The first variable on the right-hand side is the "status" indicator used in Surv().
coxsnellres <- SDLdata$status - resid(lat.m1, type="martingale")

## Then use Nelson-Aalen method to estimate the cumulative hazard function for residuals.
fitres <- survfit(coxph(Surv(coxsnellres, SDLdata$status) ~ 1,
          method='breslow'), type='aalen')
jpeg(file= "/Users/ /latency.goodness.fit.revisedorder.jpg",
    height=6, width=6, units = "in", res = 500)
plot(fitres$time, -log(fitres$surv), type='s',
          xlab='Cox-Snell Residual',
          ylab='Estimated Cumulative Hazard Function',
          main="Residual Goodness of Fit for Latency to Detection",
          bty="n", lwd=3, col="grey50")
abline(0, 1, col='red', lty=2, lwd=3)
graphics.off()

## Plot nonparametric survival curves ignoring experimental order. Superimpose fitted survival
curves
## from prop. hazards model, modified using expressions to make the curves marginal to order.

## Smoothness parameter for spline smoother.
my.spar <- 0.75
my.lwd <- 3

## MAKE GRAPH
jpeg(file= "/Users/ /latencyplot.revisedorder.jpg",
    height=6, width=8, units = "in", res = 500)
plot(survfit(Surv(latency, status) ~ experiment, data=SDLdata),
    lwd=my.lwd, bty="n",
    col=c("gray60", "deepskyblue2", "limegreen", "darkblue", "darkgreen"),
    xlab="Latency to Look (sec)", ylab="Proportion Unresponsive")
legend( "topright",
          c("Control", "2.5cm Feather", "2.5cm Snake", "Entire Feather", "Entire Snake"),
```

```
        text.col=c("gray30", "deepskyblue2", "limegreen", "darkblue", "darkgreen"),
        bty="n" )

## First generate predictions for Control and order = 0.
pred.frame <- data.frame(latency = 1:(max(SDLdata$latency)), status=1,
        experiment = "Control", revised.order = 0)

## Prediction using type="expected".
baseline.preds <- predict(lat.m1, newdata=pred.frame,
        type="expected", se.fit=FALSE)

## See documentation for predict.coxph. Survival probability is exp(-expected).
baseline.surv <- exp(-baseline.preds)

## Estimated survival curve for Control, marginal to order.
baseline.marg.surv <- baseline.surv^(0.5 * (1 + exp(coef(lat.m1)["revised.order"])))
lines(smooth.spline(pred.frame$latency, baseline.marg.surv, spar=my.spar),
        col="gray60", lwd=my.lwd)

## Estimated survival curve for 1in Feather, marginal to order.
## The expression is a modification of baseline.surv.
one.in.Feath.marg.surv <- baseline.surv^(0.5 *
        exp(coef(lat.m1)["experiment1in.Feather"]) *
        (1 + exp(coef(lat.m1)["revised.order"] +
                coef(lat.m1)["experiment1in.Feather:revised.order"])))

## The curve's support only extends to the largest latency for 1in Feather.
max.lat <- max(SDLdata$latency[SDLdata$experiment=="1in.Feather"])
lines(smooth.spline(pred.frame$latency[pred.frame$latency <= max.lat],
        one.in.Feath.marg.surv[pred.frame$latency <= max.lat], spar=my.spar),
        col="deepskyblue2", lwd=my.lwd)

## Estimated survival curve for 1in Snake, marginal to order.
one.in.Snake.marg.surv <- baseline.surv^(0.5 *
        exp(coef(lat.m1)["experiment1in.Snake"]) *
        (1 + exp(coef(lat.m1)["revised.order"] +
                coef(lat.m1)["experiment1in.Snake:revised.order"])))

## The curve's support extends to the largest latency for 1in Snake plus 25.
max.lat <- max(SDLdata$latency[SDLdata$experiment=="1in.Snake"]) + 25
lines(smooth.spline(pred.frame$latency[pred.frame$latency <= max.lat],
        one.in.Snake.marg.surv[pred.frame$latency <= max.lat], spar=my.spar),
        col="limegreen", lwd=my.lwd)
```

```
## Estimated survival curve for Full Feather, marginal to order.
Full.Feath.marg.surv <- baseline.surv^(0.5 *
        exp(coef(lat.m1)["experimentFullFeather"]) *
        (1 + exp(coef(lat.m1)["revised.order"] +
                coef(lat.m1)["experimentFullFeather:revised.order"])))

## The curve's support only extends to the largest latency for FullFeather plus 25.
max.lat <- max(SDLdata$latency[SDLdata$experiment=="FullFeather"]) + 25
lines(smooth.spline(pred.frame$latency[pred.frame$latency <= max.lat],
        Full.Feath.marg.surv[pred.frame$latency <= max.lat], spar=my.spar),
        col="darkblue", lwd=my.lwd)

## Estimated survival curve for Full Snake, marginal to order.
Full.Snake.marg.surv <- baseline.surv^(0.5 *
        exp(coef(lat.m1)["experimentFullSnake"]) *
        (1 + exp(coef(lat.m1)["revised.order"] +
                coef(lat.m1)["experimentFullSnake:revised.order"])))

## The curve's support only extends to the largest latency for FullSnake plus 25.
max.lat <- max(SDLdata$latency[SDLdata$experiment=="FullSnake"]) + 25
lines(smooth.spline(pred.frame$latency[pred.frame$latency <= max.lat],
        Full.Snake.marg.surv[pred.frame$latency <= max.lat], spar=my.spar),
        col="darkgreen", lwd=my.lwd)

graphics.off()


### SECTION 3: CALCULATE AND DISPLAY CONTRASTS FOR BOTH MODELS

## Visualization of the eight planned contrasts.
## Create a data frame of the average marginal contrasts and confidence intervals.
contrast.results <- data.frame(
  latency.contrast=rep(0,4),
  latency.ll=rep(0,4),
  latency.ul=rep(0,4),
  lookduration.contrast=rep(0,4),
  lookduration.ll=rep(0,4),
  lookduration.ul=rep(0,4),
  row.names = c("Full Snake v. Full Feather", "1in. Snake v. 1in. Feather", "Full Snake v. 1in.
Snake",
         "Full Feather v. 1in. Feather")
)

comparisons <- data.frame(
```

```
eff1=c("experimentFullSnake","experiment1in.Snake","experimentFullSnake","experimentFullF
eather"),

eff2=c("experimentFullFeather","experiment1in.Feather","experiment1in.Snake","experiment1
in.Feather")
)

## Calculate the Gaussian (0,1) quantile for 8 tests using the Bonferroni correction.
q.bonf8 <- qnorm(p=1-(.025/8), mean=0, sd=1)

## Loop over the paired comparisons for each model. Calculate the contrasts on the log scale
along
## with standard error and upper and lower confidence bounds.
for(exp.pair in 1:4){
  estimate.lat <- ave.marginal.contrast(lat.m1, as.character(comparisons[exp.pair, "eff1"]),
                                        as.character(comparisons[exp.pair, "eff2"]))
  se.lat <- se.marginal.contrast(lat.m1, as.character(comparisons[exp.pair, "eff1"]),
                                 as.character(comparisons[exp.pair, "eff2"]))
  ll.lat <- estimate.lat - q.bonf8 * se.lat
  ul.lat <- estimate.lat + q.bonf8 * se.lat
  estimate.lookduration <- ave.marginal.contrast(lookdur.m1,
as.character(comparisons[exp.pair, "eff1"]),
                                        as.character(comparisons[exp.pair, "eff2"]))
  se.lookduration <- se.marginal.contrast(lookdur.m1, as.character(comparisons[exp.pair,
"eff1"]),
                                 as.character(comparisons[exp.pair, "eff2"]))
  ll.lookduration <- estimate.lookduration - q.bonf8 * se.lookduration
  ul.lookduration <- estimate.lookduration + q.bonf8 * se.lookduration
  contrast.results[exp.pair, 1:3] <- c(estimate.lat,ll.lat,ul.lat)
  contrast.results[exp.pair, 4:6] <- c(estimate.lookduration,ll.lookduration,ul.lookduration)
}

## MAKE GRAPH
## Graphical parameters.
y.vals <- c(0.5,1.5,2.5,3.5)
my.pt.cex <- 2
my.line.wd <- 5
my.line.cl <- "gray80"
my.pt.col <- "gray80"
my.text.cex <- 1.5

jpeg(file= "/Users/ /contrastresults.revisedorder.jpg",
     height=6, width=8, units = "in", res = 500)
```

```
## Multi-panel formatting.
par(mar=c(4,1,3,1)+0.1, oma=c(2,2,5,2)+0.1)
my.layout <- layout(matrix(c(1:3), nrow=1, ncol=3, byrow=TRUE),
                widths=c(1,2,2),
                respect=FALSE)

## The left hand plot shows the contrasts.
plot(x=c(0,1), y=c(0,4), type="n", xaxt="n", yaxt="n", bty="n", xlab=" ", ylab=" ")
text(x=rep(0.5,4), y=y.vals, cex=1.5, font=2, c("Entire Snake\nvs\nEntire Feather","2.5cm
Snake\nvs\n2.5cm Feather",
                            "Entire Snake\nvs\n2.5cm Snake", "Entire Feather\nvs\n2.5cm Feather"))

## The middle plot shows the intervals for latency.
xmin <- min(contrast.results$latency.ll)
xmax <- max(contrast.results$latency.ul)
plot(x=c(xmin,xmax), y=c(0,4), type="n", xaxt="n", yaxt="n", bty="n", xlab="Ratio of Hazards",
ylab=" ",
    cex.lab=my.text.cex)
xfrom <- contrast.results$latency.ll
xto <- contrast.results$latency.ul
abline(v=0, lty=2, col="gray80", lwd=1.5)
segments(x0=xfrom,
       y0=y.vals,
       x1=xto,
       y1=y.vals,
       col=my.pt.col,
       cex=my.pt.cex,
       lwd=my.line.wd)
title(main="Latency to Look", cex.main=2, line=1.9)
points(x=contrast.results$latency.contrast, y=y.vals, pch=21, bg="gray60", cex=my.pt.cex)

## The axis is on the log scale, but the tick marks reflect the scale of measurement.
axis(side=1, at=c(log(1/3),log(1/1),log(3/1),log(10/1)), labels=c("1:3","1:1","3:1","10:1"),
    cex.axis=my.text.cex)

## The right hand plot shows the intervals for look duration.
xmin <- min(c(contrast.results$lookduration.ll), log(1/3))
xmax <- max(contrast.results$lookduration.ul)
plot(x=c(xmin,xmax), y=c(0,4), type="n", xaxt="n", yaxt="n", bty="n", xlab="Ratio of Durations",
ylab=" ",
    cex.lab=my.text.cex)
xfrom <- contrast.results$lookduration.ll
xto <- contrast.results$lookduration.ul
```

```
abline(v=0, lty=2, col="gray80", lwd=1.5)
segments(x0=xfrom,
        y0=y.vals,
        x1=xto,
        y1=y.vals,
        col=my.pt.col,
        cex=my.pt.cex,
        lwd=my.line.wd)
title(main="Duration of Looking", cex.main=2, line=1.9)
points(x=contrast.results$lookduration.contrast, y=y.vals, pch=21, bg="gray60", cex=my.pt.cex)

## The axis is on the log scale, but the tick marks reflect the scale of measurement.
axis(side=1, at=c(log(1/3),log(1/1),log(3/1),log(10/1)), labels=c("1:3","1:1","3:1","10:1"),
    cex.axis=my.text.cex)

graphics.off()

## Clean up.
rm(list=ls())
```
