# Supplementary material for: Titi monkey neophobia and visual abilities allow for fast responses to novel stimuli
Source: Sci Rep. 2021 Jan 28;11:2578. doi: 10.1038/s41598-021-82116-4 (PMC7844259; doi:10.1038/s41598-021-82116-4)
Supplement: Supplementary file 4 — Supplementary Information 4. [file 41598_2021_82116_MOESM4_ESM.pdf]

Supplemental Material 1 for Lau et al., Titi monkey neophobia and visual abilities allow for fast responses to novel stimuli

Allison R. Lau[1,2,3,*], Mark N. Grote[4], Madison E. Dufek[2], Tristan J. Franzetti[2,5], Karen L. Bales[1,2,3], & Lynne A. Isbell[1,4]

[1]Animal Behavior Graduate Group, University of California, Davis, Davis, CA, 95616, USA

[2]California National Primate Research Center, University of California, Davis, Davis, CA, 95616, USA

[3]Department of Psychology, University of California, Davis, One Shields Avenue, Davis, CA, 95616, USA

[4]Department of Anthropology, University of California, Davis, One Shields Avenue, Davis, CA, 95616, USA

[5]Present Address: Department of Biology, Duke University, Durham, NC, 27708, USA

[*]corresponding author

Allison Lau

alljones@ucdavis.edu

Estimation of Marginal Contrasts

Responses to experimental stimuli in the crossover design can be contrasted in a manner that is, broadly speaking, indifferent to the order of presentation, by *marginalizing* stimulus effects over order. We estimated marginal contrasts using the full model containing all experimental effects, described here for the response variable *latency to look*. If $\lambda(t; s, o)$ is the *hazard* at time *t*, for stimulus *s,* presented in order *o*, then the Cox Proportional Hazards model (see *e.g.* Kalbfleisch and Prentice, 2002, Chapter 4) is

$$log\ \lambda(t; s, o) = log\ \lambda^*(t) + \beta_s + \phi_o + \theta_{s,o} \quad (1)$$

where $\lambda^*(t)$ is the baseline hazard, β_s is the effect of stimulus s, ϕ_o is the effect of order o and $\theta_{s,o}$ is the interaction of stimulus s and order o.

Marginalizing the hazard for stimulus *s* over order produces a weighted average of hazards,

$$\lambda(t;s) = q\lambda(t;s,o = \text{feather first}) + (1-q)\lambda(t;s,o = \text{feather second}),$$

in which we set $q = 1/2$ for equal weights on order of presentation. The marginal hazard using equation 1 is then

$$\lambda(t;s) = (1/2)\ \lambda^*(t)[\ exp\{\beta_s + \phi_o + \theta_{s,o}\} + exp\{\beta_s + \phi_{o'} + \theta_{s,o'}\}\ ] \quad (2)$$

where *o* and *o'* correspond to feather first and feather second, respectively, and $\phi_{o'} = \theta_{s,o'} = 0$ for model identification. The marginal log hazard-ratio for two stimuli, *r* and *s*, is subsequently

$$R(r,s) = log\frac{\lambda(t;r)}{\lambda(t;s)} = \beta_r + log(1 + e^{\phi_o+\theta_{r,o}}) - [\beta_s + log(1 + e^{\phi_o+\theta_{s,o}})]\,.$$

Estimation of $R(r, s)$ proceeds by substituting estimated effects $\hat{\beta}_r$, $\hat{\beta}_s$, $\hat{\phi}_o$, $\hat{\theta}_{r,o}$, $\hat{\theta}_{s,o}$ from the fitted survival/event time model in the equation above. We obtained an approximate standard error for $R(r, s)$ by "delta method" calculations (see, *e.g.,* Stuart and Ord 1994, sections 10.5-10.7) implemented in R Language scripts available for download.

We obtained marginal survival functions for the experimental stimuli from marginal hazards (equation 2). With $\phi_{o'} = \theta_{s,o'} = 0$, equation 2 is

$$\lambda(t;s) = (1/2)\ \lambda^*(t)\ e^{\beta_s}\ (1 + e^{\phi_o+\theta_{s,o}}),$$

and the corresponding marginal survival function is

$$F_s(t) = exp\Big\{ -(1/2)\ e^{\beta_s}\ (1 + e^{\phi_o+\theta_{s,o}}) \int_0^t \lambda^*(u)\ du \Big\} = [F^*(t)]^{(1/2)\ e^{\beta_s}\ (1+e^{\phi_o+\theta_{s,o}})},$$

where $F^*(t)$ is the baseline survival function, corresponding to the control condition, with feather presented second, for model identification.

Turning to the second response variable, suppose that Y is a *duration of looking*, and

$$\lambda_{s,o} = \mathbb{E}[Y \mid s, o] = \sum_{y} y p(y \mid s, o)$$

is its expected value for stimulus s, presented in order o. The full negative binomial model has the form

$$log \lambda_{s,o} = \mu + \beta_s + \phi_o + \theta_{s,o}.$$

Marginalizing over order leads to the expression

$$\mathbb{E}[Y \mid s] = \sum_{y} y \; [q p(y \mid s, o) + (1 - q) p(y \mid s, o')] = q \; exp\{\mu + \beta_s + \phi_o + \theta_{s,o}\} + (1 - q) \; exp\{\mu + \beta_s + \phi_{o'} + \theta_{s,o'}\},$$

in which we set $\phi_{o'} = \theta_{s,o'} = 0$ for model identification, and q = 1/2, as for *latency to look*. The log ratio of expected *durations of looking* for two stimuli, r and s, is subsequently

$$log \frac{\mathbb{E}[Y \mid r]}{\mathbb{E}[Y \mid s]} = \beta_r + log(1 + e^{\phi_o + \theta_{r,o}}) - [\beta_s + log(1 + e^{\phi_o + \theta_{s,o}}],$$

formally the same as $R(r, s)$ for *latency to look*. Estimation of marginal contrasts based on the model for *duration of looking* then proceeds as for *latency to look.*

Goodness of Fit of the Models

To check the fit of the full model for *latency to look*, we examined a graph of the cumulative hazard function of the Cox-Snell residuals (as in Figure 11.1 on page 356; Klein & Moeschberger, 2005). The graph is shown as Figure S.1 below. A comparison of the cumulative hazard with the dashed, red 45-degree line suggests that the model fits well.

To check the fit of the full model for *duration of looking*, we examined a graph of the quantiles of the squared Pearson residuals from the model, *versus* corresponding quantiles of a Chi-squared distribution on one degree of freedom. The graph is shown as Figure S.2 below. Although a few large residuals are present, the model fits reasonably well.

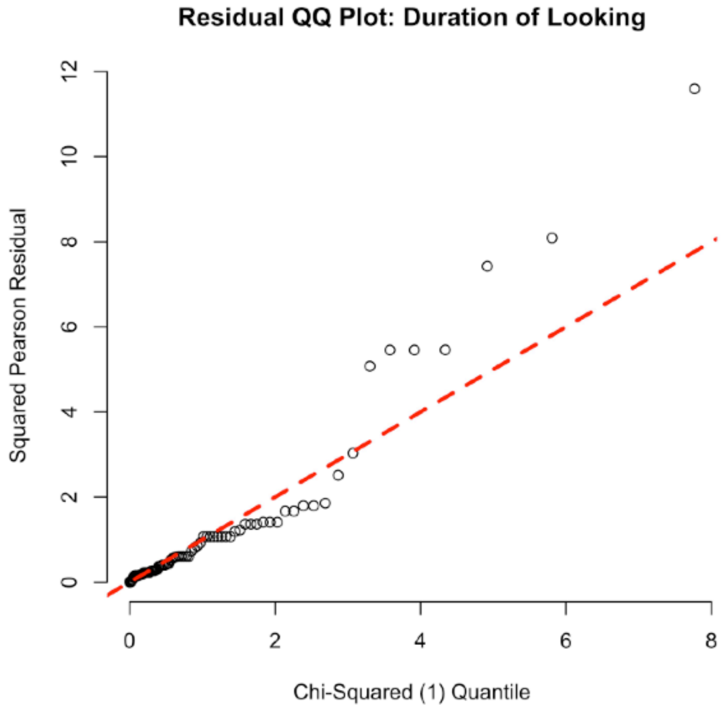

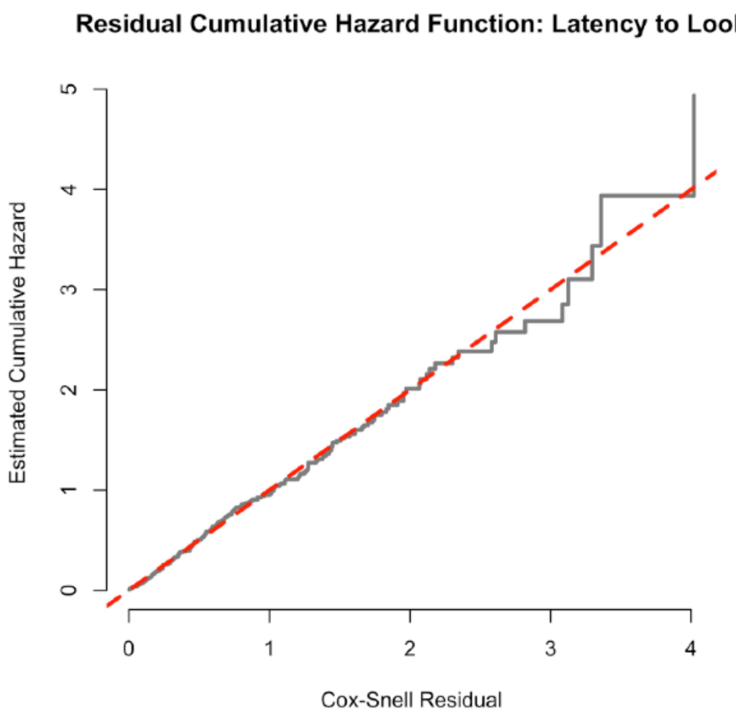

Figure S.1: Cumulative hazard function of the Cox-Snell residuals from the model for *latency to look*, along with a 45-degree line.

Figure S.2: Quantile-quantile plot, comparing squared Pearson residuals from the full model for *duration of looking* with quantiles of the Chi-squared distribution on one degree of freedom.

References

Kalbfleisch, J. D. & Prentice, R. L. (2002) The Statistical Analysis of Failure Time Data, 2nd edition. John Wiley & Sons, Inc. Hoboken, New Jersey.

Klein, J. P. & Moeschberger, M. L. (2005). Survival analysis, 2nd edition. Springer. NewYork, NY.

Stuart, A. & Ord, J. K. (1994) Kendall's Advanced Theory of Statistics,Vol.1: Distribution Theory. Edward Arnold. London.
